# MicroRNAs Regulating Tumor and Immune Cell Interactions in the Prediction of Relapse in Early Stage Breast Cancer

**DOI:** 10.3390/biomedicines9040421

**Published:** 2021-04-13

**Authors:** Chara Papadaki, Konstantina Thomopoulou, Alexia Monastirioti, George Koronakis, Maria A. Papadaki, Konstantinos Rounis, Lambros Vamvakas, Christoforos Nikolaou, Dimitrios Mavroudis, Sofia Agelaki

**Affiliations:** 1 Laboratory of Translational Oncology, School of Medicine, University of Crete, Heraklion, Vassilika Vouton, 71003 Crete, Greece; chapapadak@uoc.gr (C.P.); monasal91@gmail.com (A.M.); papadaki_maria1@yahoo.gr (M.A.P.); mavroudis@uoc.gr (D.M.); 2 Department of Medical Oncology, University General Hospital of Heraklion, Vassilika Vouton, 71110 Crete, Greece; nadiathomopoulou@hotmail.com (K.T.); koronakisgeorge@gmail.com (G.K.); kostas@rounis.gr (K.R.); vamvakasla@yahoo.gr (L.V.); 3 Department of Biology, University of Crete, Heraklion, Vassilika Vouton, 70013 Crete, Greece; nikolaou@uoc.gr; 4 Institute of Molecular Biology and Biotechnology (IMBB), Foundation of Research and Technology (FORTH), Heraklion, Vassilika Vouton, 70013 Crete, Greece; 5 Biomedical Science Research Center “Alexander Fleming”, Institute of Bioinnovation, 16672 Athens, Greece

**Keywords:** circulating miRNAs, early breast cancer, relapse, immune surveillance, immune escape, antitumor immune response

## Abstract

MicroRNAs (miRNAs) are involved in the regulation of immune response and hold an important role in tumor immune escape. We investigated the differential expression of the immunomodulatory miR-10b, miR-19a, miR-20a, miR-126, and miR-155 in the plasma of healthy women and patients with early stage breast cancer and interrogated their role in the prediction of patients’ relapse. Blood samples were obtained from healthy women (*n* = 20) and patients with early stage breast cancer (*n* = 140) before adjuvant chemotherapy. Plasma miRNA expression levels were assessed by RT-qPCR. Relapse predicting models were developed using binary logistic regression and receiver operating curves (ROC) were constructed to determine miRNA sensitivity and specificity. Only miR-155 expression was lower in patients compared with healthy women (*p* = 0.023), whereas miR-155 and miR-10b were lower in patients who relapsed compared with healthy women (*p* = 0.039 and *p* = 0.002, respectively). MiR-155 expression combined with axillary lymph node infiltration and tumor grade demonstrated increased capability in distinguishing relapsed from non-relapsed patients [(area under the curve, (AUC = 0.861; *p* < 0.001)]. Combined miR-19a and miR-20a expression had the highest performance in discriminating patients with early relapse (AUC = 0.816; *p* < 0.001). Finally, miR-10b in combination with lymph node status and grade had the highest accuracy to discriminate patients with late relapse (AUC = 0.971; *p* < 0.001). The robustness of the relapse predicting models was further confirmed in a 10-fold cross validation. Deregulation of circulating miRNAs involved in tumor-immune interactions may predict relapse in early stage breast cancer. Their successful clinical integration could potentially address the significance challenge of treatment escalation or de-escalation according to the risk of recurrence.

## 1. Introduction

Breast cancer is the leading cause of cancer-related morbidity and death among women [1]. Despite significant improvements in surgical techniques and the postsurgical treatment approaches and surveillance, almost 20% of patients with early disease will develop metastases [1].

The metastatic process is initiated when tumor cells enter the circulation and spread to other sites where they remain in the inactive state of tumor dormancy [2]. The immunoediting concept, which describes the dynamic relation between cancer and the immune system, is based on three stages: elimination, equilibrium, and escape [3]. The elimination phase refers to the attack of innate and adaptive immune cell types to eradicate cancer cells; cancer cells that survived immune destruction may then enter the equilibrium phase where immunoediting occurs. The escape phase represents the final phase of the process, where immunologically shaped tumors may grow aggressively, become clinically apparent, and establish an immunosuppressive tumor microenvironment ultimately leading to tumor progression. In all three stages, various immune cell subsets, cytokines, micro-RNAs (miRs), exosomes, and cancer cells have been shown to participate in the process [4].

MicroRNAs (miRNAs), a class of small non-coding RNA of approximately 20–22 nucleotides in length, play a significant role in the regulation of gene transcription, by binding to the 3′ untranslated region of the target mRNA [5]. They may act either as oncogenes or tumor suppressor genes, thus regulating carcinogenesis, tumor progression, and metastasis [6]. In recent years, research has focused on identifying circulating miRNAs in the plasma or serum and other biological fluids that could potentially serve as liquid biomarkers for the diagnosis and prediction of outcome in patients with breast cancer [7].

MiRNAs have been reported to regulate the development and maintenance of immune progenitors, by regulating the differentiation of immune cells as well as the maintenance and functionality of mature immune cells [8]. Furthermore, apart from acting as oncomirs or tumor suppressors, immune modulatory miRNAs exert a pivotal role in the regulation of anti-tumor immune response by modulating the expression of a broad range of immunity-associated genes in both cancer cells and tumor infiltrating lymphocytes [7,9]. Thus, microRNAs (miRNAs) have been demonstrated to reshape the tumor microenvironment (TME) towards an immunosuppressive state by decreasing the immunogenicity of cancer cells and disabling immune effectors, ultimately promoting immune escape and consequent metastatic progression [10]. Furthermore, circulating miRNAs selectively packaged in exosomes have been shown to participate in tumor escape from immunosurveillance in melanoma by regulating immune response against the tumor [11].

Based on the above evidence, we hypothesized that circulating miRNAs may reflect the crosstalk between tumor and immune cells in cancer and hold significant implications regarding tumor progression [9]. Therefore, in the current study, we investigated the differential expression of plasma miR-10b, miR-126, miR-19a, miR-20a, and miR-155 among healthy women and patients with early stage breast cancer and evaluated their prognostic role in patients with early disease. The aforementioned miRNAs were selected on the basis of their expression in breast cancer and their demonstrated involvement in immune response and modulation of antitumor immunity.

Specifically, miR-10b and miR-20a have been found to downregulate MHC Class I chain-related A and B (MICA and MICB, respectively) ligands of the natural killer (NK) activating receptor, NKG2D, thus resulting in the suppression of innate immunity and subsequent tumor growth [12]. Mir-19a is found to participate in the polarisation of tumor-associated macrophages (TAMs) from the immune-suppressive M2-like to pro-immune M1-like phenotype [13]. MiR-126 was demonstrated to control the proliferation and function of plasmatocytoid dendritic cells (pDCs), as well as the expression of Toll-like receptor (TLR) genes, which recognise pathogens including cancer cells [14]. Additionally, miR-155 has been shown to be up-regulated in macrophages and DCs following exposure to CpG and other TLR ligands [15]. Mir-155 is a critical player in adaptive immune responses as well [8]. Finally, miR-155 enhances the cytotoxicity of CD4+ T-lymphocytes and NK cells and inhibits CD8+ T cell exhaustion [16,17,18].

## 2. Materials and Methods

### 2.1. Patients’ Characteristics and Sample Collection

Two-hundred-and-fifteen patients with early stage breast cancer received adjuvant chemotherapy at the Department of Medical Oncology, University Hospital of Heraklion (Crete, Greece) between 2004 and 2011, and available plasma were identified from patient records (Figure S1). Blood was collected after surgery and before the administration of adjuvant therapy. None of the patients had received neoadjuvant chemotherapy. Blood was also collected from 20 healthy women to serve as controls during the procedure of volunteer blood donation performed in the Blood Bank Department of the University General Hospital of Heraklion; the median age of healthy volunteers was 53 years (range 35–60). All patients and healthy donors had signed an informed consent to participate in the study, which was approved by the Ethics and Scientific Committee of the University Hospital of Heraklion (ID 2029; Crete, Greece). Clinical characteristics and follow-up information for each patient were prospectively collected. Peripheral blood from patients and healthy donors was drawn early in the morning and was collected in ethylenediamine tetraacetic acid (EDTA) tubes. In patients, blood samples were obtained before starting adjuvant therapy. Plasma was isolated within 2 h from collection by centrifugation in 2500 rpm for 15 min at 4 °C, followed by a second centrifugation in 2000× *g* for 15 min at 4 °C to remove cellular debris. Samples were kept in aliquots at −80 °C until further use. Plasma samples presenting a change of colour to pink (*n* = 28), suggesting the presence of hemolysis, or samples obtained from patients lost to follow up (*n* = 25) were not processed for further analysis (Supplementary Figure S1).

### 2.2. RNA Isolation

Total RNA from 400 μL plasma was extracted by Trizol LS (Ambion, Life Technologies, Waltham, MA, USA) as described previously [19]. Briefly, following denaturation by Trizol LS, 25 fmoles of the synthetic *C. elegans* miRNA, cel-miR-39 (Qiagen GmbH, Hilden, Germany), was added to each sample to serve as an exogenous control. After the addition of chloroform followed by centrifugation, an equal volume of 700 μL of aqueous phase from each sample was transferred to a clean eppendorf tube, and was precipitated by adding 0.7 volumes of isopropanol and 1 μL of glycogen (Qiagen, Hilden, Germany). RNA pellet was resuspended in 50 μL RNAse-free water. RNA from all samples was kept at −80 °C until further use in the subsequent cDNA synthesis step.

### 2.3. Quantitative Real-Time PCR Analysis of miRNA Expression

The synthesis of cDNA and RT-qPCR was performed using TaqMan technology according to manufacturer’s instructions and as previously described [19]. Stem-loop specific primers for each miRNA were used for reverse transcription (assays’ ID for each miRNA are provided in Supplementary Table S1; Applied Biosystems, Foster City, CA, USA) in a 5 μL reaction and then diluted at 30 μL by the addition of 0.1% diethylpyrocarbonate (DEPC) H_2_O. Each miRNA was assessed by RT-qPCR in a ViiA 7 real-time PCR system (Applied Biosystems, Foster City, CA, USA). All experiments for each assay were carried out in triplicate wells. Appropriate negative controls were used in both reverse transcription and RT-qPCR reactions, where RNA input was replaced by H_2_O and no template control was used, respectively.

The fold change (log_10_) in each miRNA expression relative to the reference gene U6 snRNA was calculated using the 2^−ΔCt^ method. The expression levels of each target miRNA relative to miRNA expressed in healthy controls were calculated using the 2^−ΔΔCt^ method [20]. The suitability of U6 snRNA as a reference gene was supported by the fact that (i) it was stably and reproducibly expressed among patients and healthy donors (Figure S2; Mann–Whitney test, *p* = 0.427) and (ii) ΔCt between target miRNAs and U6 snRNA was low, demonstrating a similar range of expression.

Samples with mean Ct >35 or not amplified for target miRNAs (*n* = 5) and samples with mean Ct >22 or Ct <20 of cel-miR-39 (*n* = 3), suggesting inefficient RNA extraction, were excluded from the statistical analysis (Supplementary Figure S1). Finally, samples with mean Ct >33 or Ct <30 of U6 snRNA (*n* = 14) were also excluded (Figure S1).

### 2.4. Statistical Analysis

Statistical analysis was performed by the statistical package of the social sciences (SPSS) software, version 22.0 (SPSS Inc., Chicago, IL, USA, accessed on 10 January 2021). Patients were divided into high and low expression groups according to the median values (above or equal and below to the median values, respectively). Differential expression was evaluated by Kruskal Wallis and Mann–Whitney test.

Receiver operating curves (ROC) were constructed and area under the curve (AUC) was calculated. The performance of each miRNA was evaluated at a specific cut-off value detected using the highest Youden’s index (sensitivity + specificity − 1) by calculating sensitivity, specificity, and positive and negative predictive value. Binary logistic regression analysis was performed to identify the best discriminating combinations among miRNAs and among miRNAs with clinicopathological parameters. A 10-fold cross-validation analysis with a 70–30 split (70% training, 30% testing data) was implemented in R using a generalized linear model for logistic regression, with recurrence/non-relapse as binary target variables (http://www.r-project.org/, accessed on 10 February 2021). Statistical significance was set at *p* < 0.05 (two-sided test).

## 3. Results

### 3.1. Patients’ Characteristics and Study Design

We evaluated 140 patients with early stage breast cancer (Figure S1) and 20 healthy volunteers. Patients’ characteristics are presented in Table 1. After a median follow-up period of 102.02 months (range, 5.57–182.26), 94 patients remained disease-free and 46 experienced relapse (Table 1). The percentage of patients with tumor size more than 5 cm, four or more infiltrated lymph nodes, and histological grade III was higher in relapsed compared with non-relapsed patients (chi-square test: 75% and 25%, *p* = 0.031; 61.3% and 38.7%, *p* < 0.001; 64.3% and 44.3%, *p* = 0.034, respectively; Table 1). The other clinicopathological features were similar between relapsed and non-relapsed patients.

For the purpose of the analysis, we divided patients with early stage breast cancer into three groups, according to the timing of recurrence: (i) patients who remained disease free during the whole follow-up period (*n* = 94); (ii) patients with early relapse, defined as relapse within two years post-treatment (≤2 years; *n* = 16); and (iii) patients with late relapse, defined as relapse at 5 years or more post-treatment (≥5 years; *n* = 17). A higher percentage of patients with estrogen receptor (ER) and/or progesterone receptor (PR) positive status was encountered among patients with late compared with early relapse (69.6 vs. 30.4; *p* = 0.002 and 68.25 vs. 31.8%; *p* = 0.009, respectively). The remaining clinicopathological characteristics were similar among the two groups (Table 2).

### 3.2. Differential Expression of miRNAs among Healthy Women and Breast Cancer Patients

**Figure 1:**
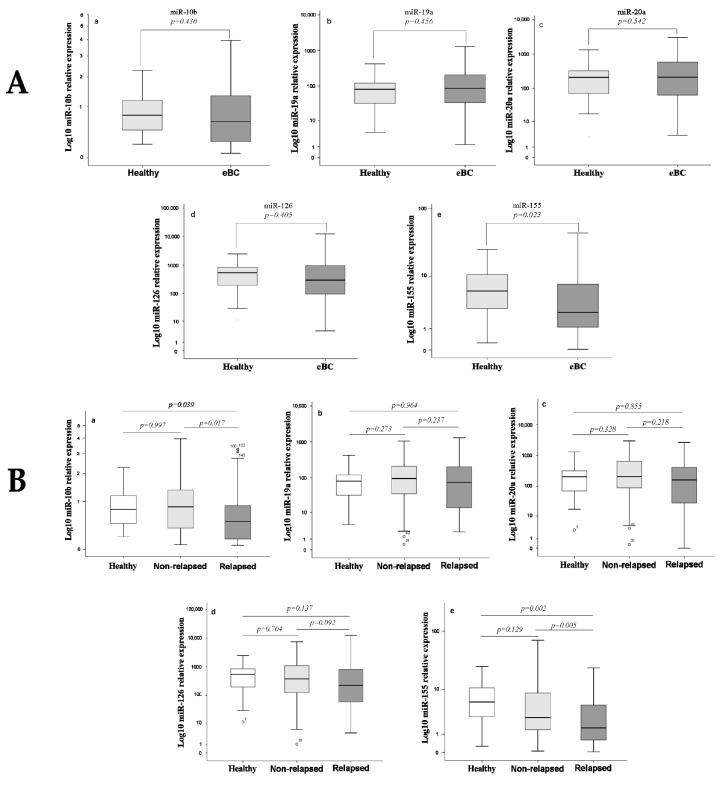
Differential expression of miRNAs in the plasma of healthy women and early stage breast cancer patients. (**A**) Fold change of miRNA expression in the plasma of healthy and whole group of early stage breast cancer patients and (**B**) of healthy, non-relapsed, and relapsed patients. (a) MiR-10b, (b) miR-19a, (c) miR-20a, (d) miR-126, and (e) miR-155 expression levels relative to U6 snRNA were assessed by the 2^−ΔCt^ method. Mann Whitney test was used to determine statistical significant differences in the expression among healthy and early stage breast cancer patients. Kruskal Wallis followed by Mann Whitney test was used to determine statistical significant differences in the expression among healthy, non-relapsed, and relapsed patients. Horizontal line on box plot depicts median and the length of the boxes is the interquartile range, representing values between the 75th and 25th percentiles of individual fold change expression values. eBC, early stage breast cancer. Statistical significance was set at *p* < 0.05.

We first compared the fold change of expression of the five miRNAs relative to U6 snRNA in the plasma of healthy donors and early stage breast cancer patients. Among the examined miRNAs, only miR-155 was differentially expressed in early stage breast cancer compared with healthy donors (Figure 1A). In particular, lower expression levels of miR-155 were observed in patients compared with healthy donors (Mann Whitney test, *p* = 0.023; Figure 1A). However, the diagnostic accuracy of miR-155 was low (Figure S3). When we compared the fold change of expression among healthy donors and patients according to relapse status, no differences were revealed in miRNAs’ expression between healthy donors and non-relapsed patients. In contrast, the expression levels of miR-10b and miR-155 were lower in patients who subsequently relapsed compared with healthy women (Mann Whitney test, *p* = 0.039 and *p* = 0.002, respectively) (Figure 1B).

### 3.3. Differential Expression of miRNAs among Relapsed and Non-Relapsed Patients

**Figure 2:**
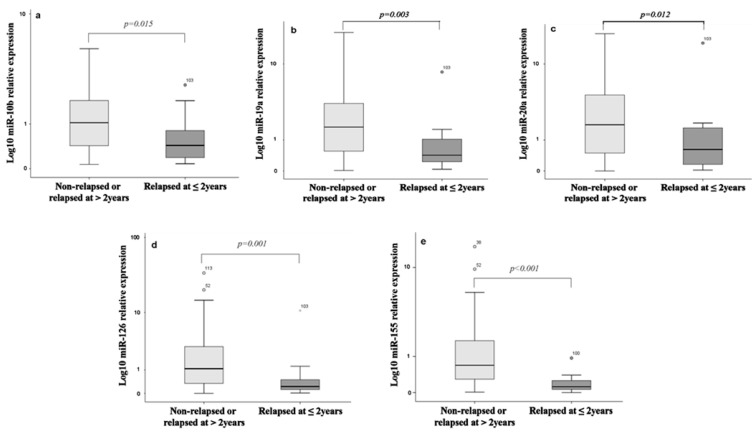
Fold change of miRNA expression in relapsed at ≤2 years compared with non-relapsed or relapsed at >2 years. The expression levels in plasma of (**a**) miR-10b, (**b**) miR-19a, (**c**) miR-20a, (**d**) miR-126, and (**e**) miR-155 were evaluated by RT-qPCR and assessed by the 2^−ΔΔCt^ method. Statistically significant differences were determined using the Mann–Whitney test and are represented by box plots. Horizontal line depicts the median expression value and the length of the boxes is the interquartile range that includes values between the 75th and 25th percentiles of individual fold change in the expression values. Statistical significance was set at *p* < 0.05.

The expression levels of the five miRNAs were compared between (i) patients who relapsed (*n* = 46) and those who did not relapse during the follow-up period (*n* = 94), (ii) patients with early relapse (≤2 years; *n* = 16) and those who either relapsed later than 2 years (*n* = 30) or remained disease free during follow-up (*n* = 94), and finally (iii) those who experienced late relapse (≥5 years; *n* = 17) and those without relapse (*n* = 94) during the whole follow-up period. The expression levels of miR-10b and miR-155 were lower in relapsed compared with non-relapsed patients (Mann Whitney test, *p* = 0.0017 and *p* = 0.005, respectively) (Figure 1B); no significant differences were revealed in the median expression levels of the other investigated miRNAs (*p* > 0.05; Figure 1B).Regarding miRNA expression according to the timing of relapse, the expression levels of all investigated miRNAs were lower in patients experiencing early relapse compared with those who either relapsed later than 2 years or remained disease free during follow-up (Figure 2). In contrast, no significant differences were found in the expression of the investigated miRNAs between patients who experienced late relapse compared with those without relapse (Mann Whitney test, *p* > 0.05).

### 3.4. Performance of miRNAs in Predictive Models

We further used miRNAs’ expression profiles to evaluate their integrative performance within relapse prediction models. To this end, we employed binary logistic regression by assessing combinations of the expression levels of miRNAs and of common clinicopathological parameters to construct the corresponding ROC curves.

**Figure 3:**
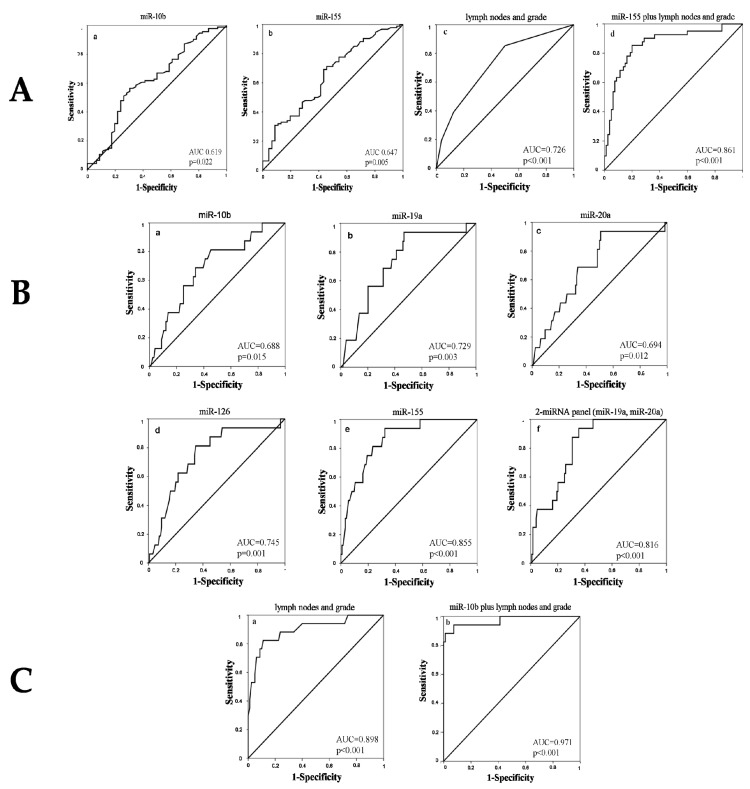
Receiver operating curves (ROC) analysis depicting the ability of plasma miRNAs alone or in combination with clinicopathological parameters to predict (panel **A**) relapse versus non-relapse, (panel **B**) early relapse (≤2 years), and (panel **C**) late relapse (≥5 years) in early breast cancer. In panel (**A**), ROC curve analysis for (a) miR-10b, (b) miR-155, and (c) clinicopathological parameters alone, or (d) in combination of miR-155 with clinicopathological parameters and their ability to predict relapsed from non-relapsed patients. In panel (**B**), ROC curve analysis of (a) miR-10b, (b) miR-19a, (c) miR-20a, (d) miR-126, and (e) miR-155 alone or (f) in a combined 2-miRNA panel and their ability to predict patients with early relapse (defined at ≤2 years). In panel (**C**), combined ROC curve analysis for (a) clinicopathological parameters alone or (b) miR-10b in combination with clinicopathological parameters and their ability to predict patients with late relapse (defined at ≥5 years). AUC, area under the curve.

ROC curve analysis for miR-10b and miR-155 alone had an AUC of 0.619 ((95% confidence interval (CI): 0.517–0.721; *p* = 0.022) (sensitivity: 69.65% and specificity: 56.4%)) and 0.647 ((95% CI: 0.550–0.745; *p* = 0.005) (sensitivity: 56.5% and specificity: 69.1%)) for the prediction of relapse, respectively (Table 3 and Figure 3A). The positive (PPV) and negative predictive value (NPV) were 43.8% and 79.1% for miR-10b and 46.3% and 75.6% for miR-155, respectively. Binary logistic regression analysis revealed that the combination of miR-155 expression along with axillary lymph node infiltration and tumor grade had superior discriminatory capability for the prediction of relapse versus non-relapse; AUC was 0.861 with sensitivity and specificity of 85.4% and 80.0%, respectively (95% CI: 0.788–0.935; *p* < 0.001) (Table 3, Figure 3A). The predictive values for the combined model of relapse were improved, with PPV and NPV being 80.6% and 82.2%, respectively.

MiR-155 and miR-19a alone had the highest performance among the investigated miRNAs in discriminating patients with early relapse with an AUC of 0.855 ((95% CI: 0.722–0.939; *p* < 0.001) (sensitivity: 93.8% and specificity: 64.5%)) and an AUC of 0.729 ((95% CI: 0.608–0.850; *p* = 0.003) (sensitivity: 93.8% and specificity: 53.2%)), respectively (Table 3, Figure 3B). The PPV and NPV were 19.4% and 97.1% for miR-19a and 27.3% and 98.8% for miR-155, respectively. However, when we assessed the combinations of miRNAs, it was shown that the combined expression of both mir-19a and miR-20a increased the accuracy in the prediction of early relapse (AUC = 0.816, 95% CI: 0.732–0.900; *p* < 0.001, sensitivity 93.8% and specificity 64.5%) (Table 3 and Figure 3B). Furthermore, the combination of miR-19a and miR-20a resulted in improved predictive values—50.0% and 89.1% for PPV and NPV, respectively. None of the clinicopathological parameters added further value in the prediction of early relapse.

Although the expression of each single miRNA was not predictive of late relapse, binary logistic regression showed that miR-10b in combination with the clinical parameters, axillary lymph node status and grade, further increased the accuracy of predicting late relapse compared with that provided by clinical parameters alone. Specifically, axillary lymph node status and grade demonstrated an AUC of 0.898 (95% CI: 0.806–0.990; *p* < 0.001) and sensitivity of 82.4%, specificity of 88.8%, PPV of 69.2%, and NPV of 90.5% in the prediction of late relapse (Table 3 and Figure 3C). Importantly, the combination of miR-10b with the respective clinicopathological characteristics demonstrated an AUC of 0.971 (0.923–1.000; *p* < 0.001), sensitivity of 88.2%, and specificity of 98.8%, respectively, in the prediction of late relapse (Table 3 and Figure 3C). Furthermore, the combined model resulted in improved PPV and NPV of 93.7% and 97.5%, respectively (Table 3 and Figure 3C).

To further validate the accuracy of the above relapse predictive models, a 10-fold cross validation was implemented in R by applying 13 different feature combinations of miRNAs and clinicopathological parameters. Mean AUC values were calculated for each 10-fold cross-validation and compared to the AUC calculated from our initial regression analysis (Table 3). No significant differences in the values of AUC in any feature combinations were observed, indicating that the performance of these models is robust and can be generalized to independent datasets (Table 3).

## 4. Discussion

We herein investigated the differential expression of miRNAs involved in the modulation of immune response in the plasma of healthy women and patients with early stage breast cancer and explored their value in the prediction of relapse. We found that miR-155 expression levels were significantly lower in patients compared with healthy blood donors, whereas miR-155 and miR-10b expressions were lower in patients who subsequently relapsed compared with healthy individuals. Plasma miR-155 along with axillary lymph node status and tumor grade demonstrated the highest accuracy in distinguishing patients who relapsed from those who did not relapse during the whole follow-up period. Regarding the timing of relapse, a 2-miRNA panel consisting of miR-19a and miR-20a had the highest performance in distinguishing those with early relapse, whereas miR-10b in combination with axillary lymph node status and grade demonstrated significant accuracy and specificity in predicting late relapse. Finally, the predictive performance of the aforementioned miRNAs was confirmed via cross validation.

Circulating miRNAs are considered to be actively secreted by the tumor and delivered to recipient cells to mediate intercellular communication [11,21]. However, the source of plasma miRNAs represents a matter of debate, with several studies suggesting that they only represent tumor byproducts resulting from tumor cell death and lysis [22]. On the other hand, other reports support that a significant proportion of circulating miRNAs may originate from immune cells in the blood and/or from those residing in the TME and potentially reflect the host’s response to the presence of the tumor [23,24]. Although the biological role of circulating miRNAs has not been clarified as yet, they have been suggested to reflect the complex interactions between tumor and immune cells throughout tumor immune escape and metastasis [25]. Body fluids typically contain free circulating miRNAs, miRNAs complexed with proteins, as well as miRNAs packed into extracellular vesicles (EVs) and, in this work, we investigated the prognostic significance of miRNAs related to immune cell function when assessed in the plasma of patients with early stage breast cancer.

Mir-155 was one of the first miRNAs identified for its regulatory role in the homeostasis and function of different immune cell subsets [25,26]. Importantly, miR-155 is considered as a critical molecule causing macrophage M1 polarization and its over-expression was shown to re-program the anti-inflammatory, pro-tumoral M2 to the anti-tumor M1 phenotype of TAMs [27]. As such, miR-155 has also been shown to modulate anti-tumor immune effector responses regulating genes in different immune cell subsets according to the cellular context [28]. Thus, overexpression of miR-155 in TAMs significantly decreased tumor growth and promoted apoptosis of tumor cells [27]. In addition, knock down of miR-155 in the myeloid cell compartment shifted TAMs from M1- to M2-phenotype associated with increased production of pro-tumor cytokines and accelerated tumor growth in a mouse model of spontaneous breast carcinogenesis that closely mimics tumor–host interactions seen in humans [29]. MiR-155 has been also shown to regulate adaptive immunity by promoting IFNγ anti-tumor responses by CD4+ and CD8+ T-cells [30]. Furthermore, miR-155 is required for effector CD8+ T cells’ accumulation and efficient control of tumor growth in mice models [31]. In contrast, in another report, miR-155 regulated the accumulation of MDSCs in the TME and was required by MDSCs to facilitate tumor growth [32].

MiR-155 is considered as an oncomir, with deregulated levels demonstrated in several types of cancer, including breast cancer [8,33,34], albeit its role in carcinogenesis and tumor growth remains controversial [35]. In a mouse mammary model, stable expression of miR-155 in 4T1 breast cancer cells inhibited tumor dissemination from mammary fat pads to the lung by preventing epithelial mesenchymal transition (EMT) [36]. However, the prognostic significance of miR-155 expression in the tumor tissue or the circulation has not been clarified as yet in breast cancer. Specifically, high expression of serum miR-155 has been correlated with unfavorable clinical characteristics and shorter overall survival in early stage breast cancer patients [37]. Moreover, in another cohort of early stage breast cancer patients, high serum miR-155 expression was correlated with worse disease-free survival [38]. In addition, miR-155 was increased in the serum of relapsed as compared with non-relapsed breast cancer patients [39]. In contrast, we herein show that plasma miR-155 expression levels were lower in patients compared with healthy women and that low mir-155 expression was predictive of relapse in patients with early disease. Our observations support the association of increasing plasma miR-155 expression levels with better prognosis in early stage breast cancer. Taking into account the immune regulatory role of miR-155, they further suggest that higher plasma miR-155 levels could potentially indicate the presence of an efficient antitumor immune response. Nevertheless, the prognostic significance of miR-155 merits further evaluation in breast cancer.

Mir-19a was first identified as the key oncogenic component of the miR-17-92 cluster, which is frequently amplified or over-expressed in human cancers [40] and exerts oncogenic activity by suppressing Phosphatase and tensin homolog (PTEN) [41] or by modulating the oncogenic properties of c-Myc [42]. In contrast, a favourable role has been ascribed for miR-19a in antitumor immune response. In a mouse breast cancer model, miR-19a induced the switch from the immunosuppressive M2 to the pro-inflammatory M1 TAM phenotype by targeting Fos-related antigen-1 (*Fra-1)* and the Fra-1/STAT3 signaling pathway and controlled tumor growth and invasion [13].

In the clinical setting, the up-regulation of miR-19a in triple negative breast cancer tissues [40] has been associated with chemotherapy resistance in the luminal A breast cancer subtype [43]. In addition, it was shown that miR-19a was up-regulated in the whole blood of early breast cancer patients as compared with healthy donors [44] as well as in the serum of patients with increased risk of progression [45]. In contrast, in our patient cohort, plasma miR-19a expression was lower in patients who relapsed within 2 years. Importantly, miR-19a combined with miR-20a expression yielded an AUC of 0.861 with sensitivity of 93.8% and specificity of 64.5% in the prediction of early relapse. These findings suggest that higher plasma miR-19a expression levels could also reflect favourable tumor-immune interactions associated with improved patient outcomes; however, miR-19a significance needs to be further investigated in early stage breast cancer.

Late relapse is of considerable concern among disease-free patients with breast cancer, and in the clinic, there are no accurate tools to identify patients at risk. Of note, in our study, miR-10b expression combined with the clinical information on axillary lymph node status and tumor grade yielded an AUC of 0.971 with sensitivity of 88.2% and specificity of 98.8% for the prediction of late relapse (*p* < 0.001). MiR-10b directly targets MICB, the stress-induced ligand of NKG2D receptor, expressed by tumor cells, thus impairing NK-mediated recognition and elimination of tumor cells [46]. Moreover, miR-10b has been shown to trigger tumor invasion and metastasis in xenograft models [47].

Contradictory results exist regarding the role of deregulated miR-10b expression in breast cancer. MiR-10b was found to be down-regulated in primary breast tumors compared with normal tissues [33] and, in another study, miR-10b expression was lower in early breast cancer patients who experienced relapse [48,49]. In accordance, we observed that circulating miR-10b was significantly lower in patients who relapsed compared with healthy donors as well as in patients with early relapse as compared with the remaining patients. To date, only limited information exists regarding the role of circulating miR-10b in breast cancer. In such a study, mir-10b along with miR-155 was increased in the plasma of breast cancer patients compared with healthy controls [50]; however, we did not observe any differences in plasma miR-10b expression between patients and healthy donors.

We further showed that plasma miR-20a and miR-126 expression levels were significantly decreased in patients who experienced relapse within 2 years from surgery. In accordance with our results, miR-20a expression was significantly decreased in the plasma of breast cancer patients compared with healthy controls and low expression was associated with unfavourable clinical characteristics [51]. Moreover, mir-126 is downregulated in breast cancer tumors and serum compared with normal tissue [52], and its expression is significantly lower in relapsed compared with non-relapsed patients [53]. The above evidence suggests that plasma miR-20a and miR-126 expression levels are associated with tumor progression and metastatic dissemination in breast cancer.

Controversial results exist regarding the role of miR-20a in anti-tumor immune response. In Zhang et al., miR-20a along with miR-17 alleviated the suppressive potential of myeloid derived suppressor cells (MDSCs) by modulating STAT3 expression [54]. On the other hand, increased levels of miR-20a in tumor cells suppress NK cells’ cytotoxicity by targeting the expression of the NKG2D ligands, MICA/B [55]. Besides its role as an important regulator of the innate immune response to pathogens [14], miR-126 has also been shown to modulate the TME and to suppress tumor invasion and lung metastasis of breast cancer cells [56]. Specifically, miR-126 independently suppressed the sequential recruitment of mesenchymal stem cells and inflammatory monocytes, the precursors of tumor associated macrophages, into the tumor stroma by targeting stroma cell-derived factor-1 alpha (SDF1a) or the chemokine (C-C motif) ligand 2 (CCL2) in cancer cells [56]. In the same line, Tavazoie et al. showed that miR-126 along with miR-335 suppressed lung and bone metastasis in human breast cancer xenograft models [53].

The investigated miRNAs have been reported to be involved in the regulation of innate immune responses [57] and our results show that their expression is associated with disease relapse. Cells of the innate immune system are involved in pro- or anti-tumor interactions with cancer cells and our results indicate that circulating miRNAs may hold a critical role in this decision-making process by targeting key molecules of innate immune pathways during tumor-immune interactions [25]. However, it should be noted that we cannot conclude herein neither on the function of these miRNAs nor on their association with the abundance and polarity of immune cells in the TME.

## 5. Conclusions

In summary, our results indicate that miRNAs selected according to their suggested involvement in immune cell function assessed in the plasma predict disease relapse in early stage breast cancer patients years before its clinical detection. A recent statistical framework analysis using clinical parameters and molecular data in a large cohort of breast cancer patients resulted in the modelling of the risks for loco-regional and distant relapse [58]. Our results show that circulating miRNAs may provide additive value in these prediction models. We consider that the successful integration of circulating miRNAs into clinical practice could improve patient prognostication in an effort to address the significant challenge of treatment escalation or de-escalation according to the risk of recurrence in early stage breast cancer patients. However, further comparative studies of plasma miRNAs, miRNAs derived from circulating leukocytes, or from EVs could potentially be more informative towards the identification of blood fractions with optimal diagnostic power as compared with the interrogation of plasma only miRNAs.

## Figures and Tables

**Table 1:** Characteristics of early stage breast cancer patients.

	All Patients	Non Relapse	Relapse	
Characteristic	***n* (%)**	***n* (%)**	***n* (%)**	***p-*Value**
Number of patients	140	94 (67.2)	46 (32.8)	
Age, median (range)	55 (27–82)	54 (35–79)	56 (27–82)	ns *
Menopausal status				ns *
Premenopausal	53 (37.9)	38 (40.4)	15 (32.6)	
Postmenopausal	87 (62.1)	56 (59.6)	31 (67.4)	
Tumor size (cm)				0.031 *
T1	62 (44.3)	44 (46.8)	18 (39.1)	
T2	70 (50.0)	48 (51.1)	22 (47.9)	
T3	8 (5.7)	2 (2.1)	6 (13.0)	
Grade				0.034 *
I	5 (3.6)	5 (5.3)		
II	56 (40.0)	42 (44.7)	14 (30.4)	
III	67 (47.9)	39 (41.5)	28 (60.9)	
Lobular	8 (5.7)	4 (4.3)	4 (8.7)	
Unknown	4 (2.9)	4 (4.3)		
Infiltrated lymph nodes				<0.001 *
0	60 (42.9)	49 (52.1)	11 (23.9)	
1–3	50 (35.7)	34(36.2)	16 (34.8)	
≥4	30 (21.4)	11 (11.7)	19 (41.3)	
ER status				ns *
Positive	88 (62.9)	58 (61.7)	30 (65.2)	
Negative	52 (37.1)	36(38.3)	16 (34.8)	
PR status				ns *
Positive	88 (62.9)	60(63.8)	28 (60.9)	
Negative	52 (37.1)	34 (36.2)	18 (39.1)	
Her2 status				ns *
Positive	19 (13.6)	11(11.7)	8 (17.4)	
Negative	121 (86.4)	83(88.3)	38 (82.6)	
Adjuvant chemotherapy				
Anthracyclines-based	10 (7.1)	7 (7.4)	3 (6.5)	
Taxanes + Antracyclines	95 (67.9)	59 (62.9)	36 (78.3)	
Taxanes-based	26 (18.6)	20(21.3)	6 (13)	
Other	3 (2.1)	3 (3.3)		
None	6 (4.3)	5 (5.2)	1 (2.2)	

ER, estrogen receptor; PR, progesterone receptor; HER2, human epidermal growth factor receptor 2; * Pearsons’ chi-square for relapsed and non-relapsed patients; ns, not significant.

**Table 2:** Characteristics of patients with early and late relapse.

	Early Relapse	Late Relapse	*p*-Value
Characteristic	*n* (%)	*n*	
Number of patients	16 (11.4)	17	
Age, median (range)	56 (27–82)	55 (41–74)	ns *
Menopausal status			ns *
Premenopausal	7 (43.8)	5 (29.4)	
Postmenopausal	9 (56.2)	12 (70.2)	
Tumor size (cm)			ns *
T1	5 (31.3)	8 (47.1)	
T2	9 (56.3)	7 (41.2)	
T3	2 (12.5)	2 (11.8)	
Grade			ns *
I			
II	6 (37.5)	3 (17.6)	
III	10 (62.5)	14 (82.4)	
Infiltrated lymph nodes			ns *
0	6 (37.5)	1 (5.9)	
1–3	6 (37.5)	7 (41.2)	
≥4	4 (25)	9 (52.9)	
ER status			0.002 *
Positive	7 (43.8)	16 (94.1)	
Negative	9 (56.2)	1 (5.9)	
PR status			0.009 *
Positive	7 (43.8)	15 (88.2)	
Negative	9 (56.3)	2 (11.8)	
Her2 status			ns *
Positive	3 (18.8)	4 (23.5)	
Negative	13 (81.2)	13 (76.5)	
Adjuvant chemotherapy			ns *
Anthracyclines-based	1 (6.3)	1 (5.9)	
Taxanes+Antracyclines	11 (68.7)	15 (88.2)	
Taxanes-based	3 (18.7)	1 (5.9)	
Others	1 (6.3)		

Early relapse, ≤2 years post-treatment; late relapse, ≥5 years post-treatment; ER, estrogen receptor; PR, progesterone receptor; HER2, human epidermal growth factor receptor 2; * Pearsons’ chi-square for early and late relapse.

**Table 3:** Performance of miRNAs and their combinations for the prediction of relapse in early stage breast cancer.

Potential Predictors	Cutoff	Sensitivity (%)	Specificity (%)	AUC (95% CI)	*p-*Value	PPV (%)	NPV (%)	Mean AUC	Mean *p*-Value
								(10-Fold Cross Validation)	(10-Fold Cross Validation)
**Relapse**									
miR-10b	0.920	69.6	56.4	0.619 (0.517–0.721)	0.022	43.84	79.1	0.605	0.01891
miR-155	0.405	56.5	69.1	0.647 (0.550–0.745)	0.005	46.3	75.6	0.684	0.00643
LN and grade	0.416	85.4	50.0	0.726 (0.631–0.821)	<0.001	72.7	70.0	0.699	0.00121
miR-155 and LN and grade	0.292	85.4	80.0	0.861 (0.788–0.935)	<0.001	80.6	82.2	0.767	0.00329
**Early relapse (≤2 years)**									
miR-10b	0.855	81.3	54.8	0.688 (0.556–0.819)	0.015	18.8	95.8	0.704	0.01813
miR-19a	1.560	93.8	53.2	0.729 (0.608–0.850)	0.003	19.4	97.1	0.725	0.00410
miR-20a	1.940	93.8	49.2	0.694 (0.564–0.823)	0.012	19.2	98.4	0.689	0.01011
miR-126	0.530	81.0	65.3	0.745 (0.620–0.829)	0.001	22.4	96.3	0.739	0.00082
miR-155	0.445	93.8	64.5	0.855 (0.722–0.939)	<0.001	27.3	98.8	0.860	0.00078
miR-19a, miR-20a	0.265	93.8	64.5	0.816 (0.732–0.900)	<0.001	50.0	89.1	0.834	0.00071
**Late relapse (≥5 years)**									
miR-10b	1.205	88.2	51.1	0.642 (0.516–0.768)	0.063	22.6	93.9	0.628	0.07835
LN and grade	0.115	82.4	88.8	0.898 (0.806–0.990)	<0.001	69.2	90.5	0.865	0.00089
miR-10b and LN and grade	0.467	88.2	98.8	0.971(0.923–1.000)	<0.001	93.7	97.5	0.931	0.00066

AUC, area under the curve; CI, confidence intervals; LN, lymph nodes; PPV, positive predictive value; NPV, negative predictive value. Statistical significance was set at *p* < 0.05.

## Data Availability

The data presented in this study are available on request from the corresponding author.

## Supplementary Materials

The following are available online at https://www.mdpi.com/article/10.3390/biomedicines9040421/s1, Figure S1: Flow chart of the study. Ct, cycle threshold. Figure S2: U6 snRNA expression levels between healthy donors and early stage breast cancer patients. Mann Whitney test was used to determine statistically significant differences and the results were displayed on box plots. The *p*-value is shown. Figure S3: Capability of miR-155 to distinguish healthy women from early breast cancer patients. AUC, area under curve; CI, confidence intervals. Table S1: Assay ID for each miRNA used in the study.
