# Supplementary material for: MicroRNAs Regulating Tumor and Immune Cell Interactions in the Prediction of Relapse in Early Stage Breast Cancer
Source: Biomedicines. 2021 Apr 13;9(4):421. doi: 10.3390/biomedicines9040421 (PMC8069787; doi:10.3390/biomedicines9040421)
Supplement: Supplementary file 1 [file biomedicines-09-00421-s001.pdf]

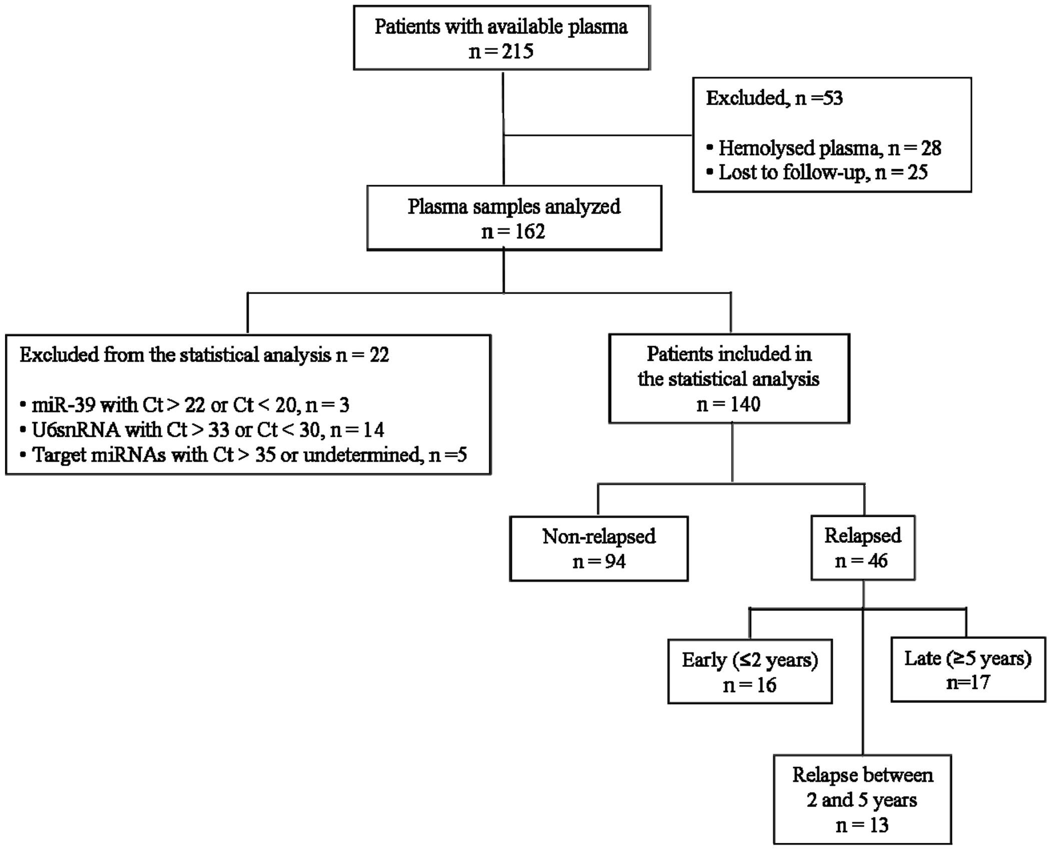

Figure S1. Flow chart of the study. Ct, cycle threshold.

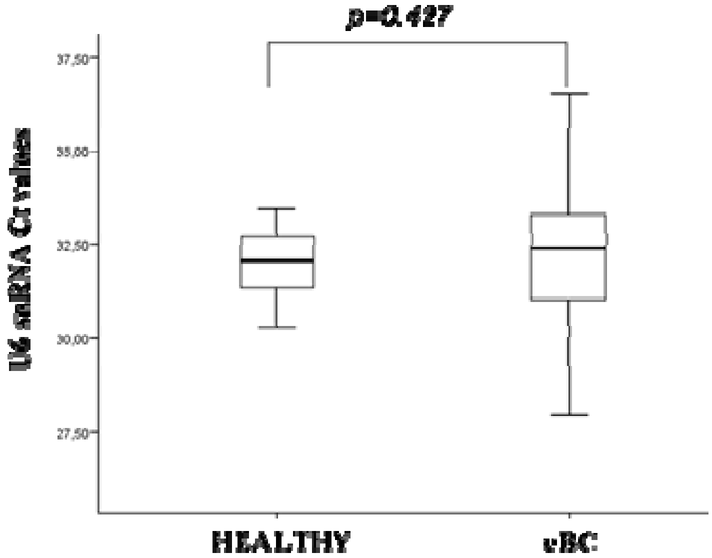

Figure S2. U6 snRNA expression levels between healthy donors and early stage breast cancer patients. Mann Whitney test was used to determine statistically significant differences and the results were displayed on box plots. The *p* value is shown.

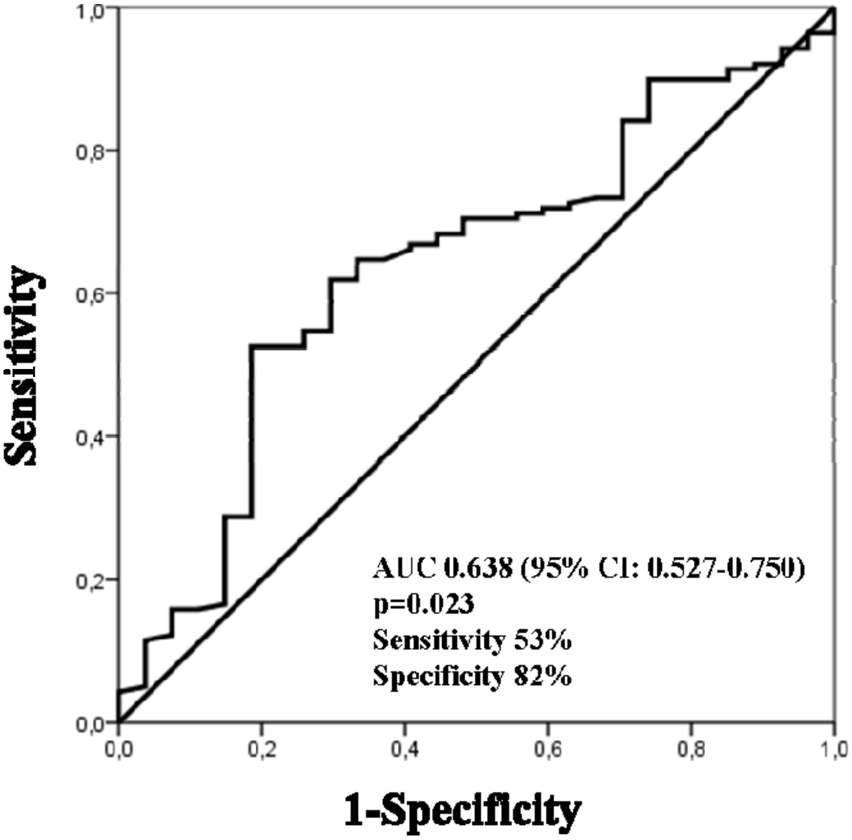

Figure S3. Capability of miR-155 to distinguish healthy women from early breast cancer patients. AUC, area under curve; CI, confidence intervals

Table S1. Assay ID for each miRNA used in the study.

**Name**	**Assay ID**
hsa-miR-10b-5p	002218
hsa-miR-19a-3p	000395
hsa-miR-20a-5p	000580
hsa-miR-126-3p	002228
hsa-miR-155-5p	002623
U6 snRNA	001973
cel-miR-39-3p	000200
